# Application of response surface methodology on the nanofluid flow over a rotating disk with autocatalytic chemical reaction and entropy generation optimization

**DOI:** 10.1038/s41598-021-81755-x

**Published:** 2021-02-17

**Authors:** Tahir Mehmood, Muhammad Ramzan, Fares Howari, Seifedine Kadry, Yu-Ming Chu

**Affiliations:** 1 School of Natural Sciences (SNS), National University of Sciences and Technology (NUST), Islamabad, Pakistan; 2 Department of Computer Science, Bahria University, Islamabad, 44000 Pakistan; 3 Department of Mechanical Engineering, Sejong University, Seoul, 143-747 Korea; 4 College of Natural and Health Sciences, Zayed University, 144543 Abu Dhabi, UAE; 5 Department of Mathematics and Computer Science, Faculty of Science, Beirut Arab University, Beirut, 115020 Lebanon; 6 Department of Mathematics, Huzhou University, Huzhou, 313000 People’s Republic of China; 7 Hunan Provincial Key Laboratory of Mathematical Modeling and Analysis in Engineering, Changsha University of Science & Technology, Changsha, 410114 People’s Republic of China

**Keywords:** Software, Mechanical engineering

## Abstract

The role of nanofluids is of fundamental significance in the cooling process of small electronic devices including microchips and other associated gadgets in microfluidics. With such astounding applications of nanofluids in mind, it is intended to examine the flow of magnetohydrodynamic nanofluid comprising a novel combination of multi-walled carbon nanotubes and engine oil over a stretched rotating disk. The concentration equation is modified by considering the autocatalytic chemical reaction. The succor of the bvp4c numerical technique amalgamated with the response surface methodology is secured for the solution of a highly nonlinear system of equations. The sensitivity analysis is performed using a response surface methodology. The significant impacts of the prominent arising parameters versus involved fields are investigated through graphical illustrations. It is observed that the skin friction coefficient and local Nusselt number are positively sensitive to nanoparticle volume fraction while it is positively sensitive to the suction parameter. It is negatively sensitive to the Magnetic parameter. The skin friction coefficient is negatively sensitive to all input parameters.

## Introduction

In numerous chemical reaction systems including food processing, combustion, catalysis, and biomedical equipment production, the role of homogeneous-heterogeneous chemical reactions is fundamental. The homogeneous chemical reaction is identified when the reactants and their outcomes are in the identical phase. Nevertheless, when these two appear in distinct phases, or the reaction occurs at the peripheral surface of the catalyst in separate phases, then reactions are identified as a heterogeneous chemical reaction. Practically, homogeneous and heterogeneous reactions are found in the biochemical processes, ignition, water, food processing, and air pollutants, and many other extensive areas. The pioneering effort was done by Chaudhary and Merkin ^1^ who studied the homogeneous and heterogeneous reactions isothermal process on the boundary of the assumed surface. The proposed model ^1^ was improved by Merkin ^2^ by considering the impact of homogeneous and heterogeneous reactions for Newtonian fluid flow over a surface. The flow of hybrid nanofluid comprising Copper, Silver, Alumina, and Titanium oxide amalgamated with ethylene glycol and water with autocatalytic chemical reactions over a rotating disk is studied by Das et al. ^3^. It is reported in this exploration that the concentration for the combination of Titanium oxide with ethylene glycol is (30–36)% dominant in comparison to the mixture of Titanium oxide with water. Hayat et al. ^4^ examined the nanofluid flow over a rotating disk with a combination of water with both types of carbon nanotubes (CNTs) under the influence of homogeneous and heterogeneous reactions. An important outcome of this study is that the fluid concentration deteriorates for the homogeneous chemical reaction. Two different nanofluid flow combinations comprising the Copper–water and Alumina–water with the impact of autocatalytic chemical reactions and melting heat transfer over a rotating disk are studied by Imtiaz et al. ^5^. The significant result of the presented model is that the surface drag force of the nanofluid in the case of Alumina-water is dominant as compared to the Copper–water combination. Some latest explorations featuring the chemical reaction aspect in varied fluid flows may be found in ^6–10^.

Numerous attempts can be witnessed in the literature in the recent past for the enhancement of the heat transport and cooling phenomenon. Adequate cooling is a must for the end product in numerous manufacturing processes like computers, power electronics, and engines, etc. Numerous applications of nanofluids may be found in heating/cooling appliances, nano-drug delivery, microelectronics, fuel cells, pharmaceutical processes, and nuclear power plants, etc. The classical technique engaged in the cooling process was the use of air. For the cooling of numerous electronic gadgets, the use of some liquid is essential whenever heat flux is more than 100 W/cm^2^. The nanofluid with enhanced heat transfer capabilities is an amalgamation of nano-sized (< 100 nm) metallic particles and some customary fluid. The nanofluids are used in numerous industrial processes including nuclear reactors, food processing, transportation, and biomedicine. Choi and Eastman ^11^ introduced the term ’nanofluid’ for the first time in 1995. Two renowned nanofluid models titled “Tiwari and Das” ^12^ and “Buongiorno” ^13^ are commonly used in the existing literature. Lately, Tassaddiq et al. ^14^ examined the hybrid nanofluid flow with CNTs of both types, and Iron oxide (magnetic ferrite nanoparticles) immersed into water over an infinite solid rotating disk. It is comprehended from this study that the fluid velocity and temperature are highly dependent upon the disk rotation speed. The flow of copper–water nanofluid flow over a rotating disk with nonlinear thermal radiation, Darcy Forchheimer, and modified Fourier law in a porous medium is elaborated by Nayak et al. ^15^. An entropy minimization analysis is also conducted here. It is comprehended here that the Reynolds number and the radiation parameter possess opposing trends for the entropy generation rate. Reddy et al. ^16^ highlighted the impacts of chemical reaction in the flow of a hybrid nanofluid comprising an Ag–Cu–water mixture in a permeable medium over a rotating disk. It is revealed in this study that fluid temperature is enhanced for both combinations. Some more studies emphasizing the nanofluid flow over a rotating disk may be found in ^4,17–30^.

The novel notion of Entropy generation minimization in a convective heat transfer process was floated by Bejan ^31^ in 1979. The idea of entropy generation is employed to boost the effectiveness of thermal engineering devices in numerous thermodynamic systems ^32^. The entropy generation is utilized to measure the molecular disorder or chaos in some thermodynamic system. The second law of thermodynamics disclosed that the quality of energy loss is in inverse proportionate relation with the molecular turmoil. It is also witnessed that the entropy generation is triggered due to the difference in temperatures in heat transfer and energy dissipation. Entropy may be found in electrical resistance, mixing of liquids, friction, chemical reactions, unstained expansion, deformation of plastics, and unnecessary transfer of heat in a finite temperature difference, and internal friction. Therefore, immense attention is paid to the improvement of heat transfer in various engineering applications. Wakeel et al. ^33^ discussed the second-grade nanofluid flow under the influence of modified Fourier law over an extended rotating disk with Hall effect and entropy generation. The salient outcome of the existing study is that the impact of the Bejan number is strengthened for numerous estimates of the temperature difference and diffusion parameter. The entropy generation minimization analysis with activation energy and binary chemical reaction impacts on a Sisko nanofluid flow over a rotating disk is studied by Ijaz et al. ^34^. It is comprehended that entropy is increased for shear-thinning fluids. Farooq et al. ^35^ examined the flow of hybrid nanofluid comprising (Cu–Al_2_O_3_)–water with impacts of viscous dissipation, and entropy generation measurement over a permeable rotating disk. The major outcome reveals that the entropy generation in the case of Al_2_O_3_–water is weaker than (Cu–Al_2_O_3_)–water combination. Some more explorations studying entropy generation over rotating disks may be found in ^36,37^.

The experimental design is a vital component in industrial and applied research. In experimental design, one or more response is measured over the experimental units, where a combination of levels of input parameters is applied over the experimental unit. For appropriate observation of the mechanism and determining the levels of input parameters that optimize the response, the response surface methodology (RSM) is a potential candidate in experimental design ^38–40^. RSM can help the researcher in developing the list of experimental designs that can be used for predicting the response. It can help to adjust the theoretical constraints to study the specific model term or interaction. Moreover, it can suggest the optimal level or value of input parameters that can optimize the response.

In the current article, the aim to investigate the model dependencies of the response variables which are skin friction coefficients and the local Nusselt number with the input parameters which are models’ parameters including suction parameter, Nanoparticle volume fraction, and Magnetic parameter. The use of RSM to determine the optimal parameter’s level is frequently observed in related studies ^41–44^. Moreover, the experimental scheme like RSM is usually linked with sensitivity analysis to investigate the dependency of response on the input parameters ^45–47^. The uniqueness of the existing model as shown in Table 1 is verified by comparing the envisioned model with the published articles.

**Table 1:** Literature survey for inimitability of the existing model.

Authors	Tiwari and Das model	Rotating disk	Autocatalytic chemical reaction	Entropy generation minimization	MWCNTs and engine oil
Tassaddiq et al. ^14^	Yes	Yes	No	No	No
Nayak et al. ^15^	Yes	Yes	No	No	No
Gholinia et al. ^28^	Yes	Yes	Yes	No	No
Hayat et al. ^4^	Yes	Yes	Yes	No	No
Present	Yes	Yes	Yes	Yes	Yes

Given the foregoing, it is revealed from the above-cited literature that abundant studies are available that discuss the nanofluid flow over a rotating disk. But no research is presented so far that studies the nanofluid flow comprising MWCNTs and engine oil amalgamation with irreversibility analysis. The uniqueness of this study is enhanced by examining the subject nanofluid flow in a different prospect by using the response surface methodology that helps us to do the sensitivity analysis. The numerical solution of the problem is also found by using the bvp4c MATLAB software function. The sensitivity analysis is performed using a response surface methodology. The graphs of pertinent parameters are also drawn to witness their behavior versus involved distributions. The unique objectives of the present exploration are to answer the subsequent salient questions:

I. Do the skin friction coefficients and the local Nusselt number models well fit the data?
II. What about the distribution of skin friction coefficients and the local Nusselt number?
III. What are the sensitive factors for modeling skin friction coefficients and the local Nusselt number?
IV. Identify the level of factors that optimizes the skin friction coefficients and the local Nusselt number?
V. How nanoparticle volume fraction affects the fluid velocity in the radial direction?

## Mathematical formulation

The assumptions of the presented model are given as under:

i. Three-dimensional viscous nanofluid flow.
ii. The fluid flow contains multi-walled carbon nanotubes (MWCNTs) and engine oil.
iii. The disk is placed at $z = 0,$ and is rotating with an angular velocity $\Omega .$
iv. The movement of the disk is taken as axisymmetric and the impact of tangential motion is overlooked.
v. The components of velocity in cylindrical coordinates are taken as $\left( {r,\theta ,z} \right).$
vi. The disk is extended in the radial direction with a velocity $u_{w} = cr,$ with a steady rate $c.$
vii. The ambient velocity is taken as $u_{e} = ar.$
viii. The temperature $T_{w}$ is at the disk surface and far away from the disk is considered as $T_{\infty } .$
ix. A magnetic field of strength $B_{0}$ is applied perpendicular to the disk.
x. The induced magnetic field is overlooked owing to our supposition of a small Reynolds number.
xi. The amalgamated nanofluid (MWCNTs)-engine oil is assumed to be in thermal balance with the no-slip condition.
xii. The impacts of homogeneous-heterogeneous reactions are considered.

**Figure 1:**
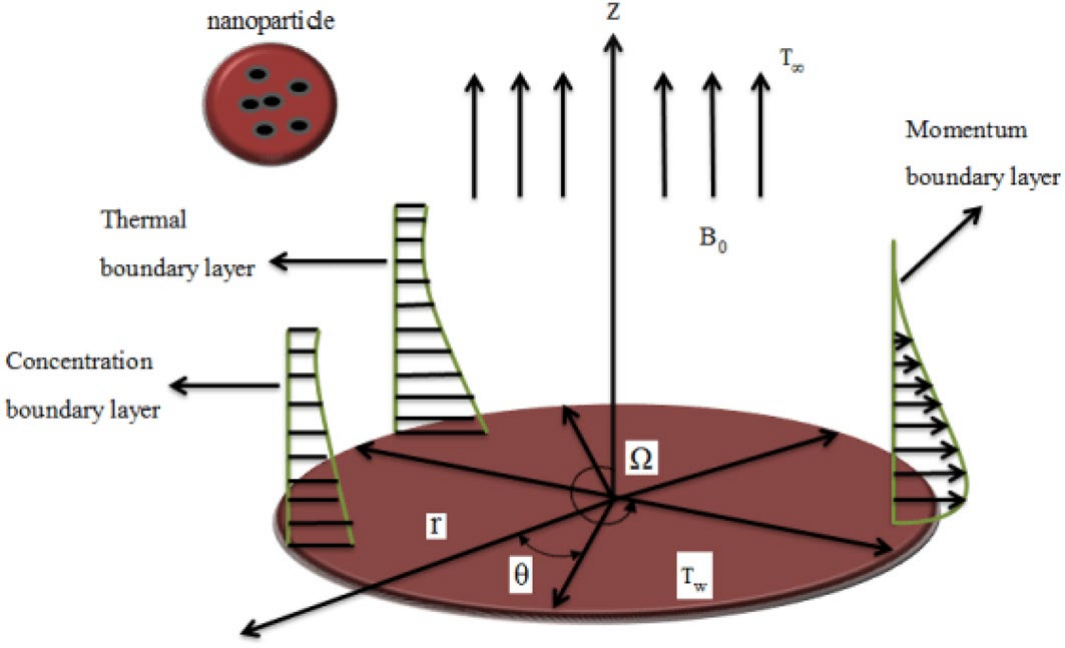
Physical flow chart.

The configuration of all the above assumptions is given in Fig. 1.

The assumption of the autocatalytic chemical reactions with chemical species $A_{1}$ and $B_{1} .$ Both homogeneous and heterogeneous chemical reactions ^1,48^ occur as follows:

1 $$ A_{1} + 2B_{1} \to 3B_{1} ,\,\,\,\,\,rate = k_{c} ab^{2} . $$

The first order isothermal reaction on the outer surface of the catalyst is taken as:

2 $$ A_{1} + B_{1} \to 2B_{1} ,\,\,\,\,\,rate = k_{s} a. $$

Both chemical reactions are considered as isothermal. The chemical species $A_{1}$ and $B_{1}$ possess $k_{c}$ and $k_{s}$ as rate constants, and $a,b$ are the respective concentrations. The governing model equations considering the aforementioned assumptions are ^4,28^:

3 $$ \frac{\partial u}{{\partial r}} + \frac{u}{r} + \frac{\partial w}{{\partial z}} = 0, $$

4 $$ u\frac{\partial u}{{\partial r}} + w\frac{\partial u}{{\partial z}} - \frac{{v^{2} }}{r} = - \frac{1}{{\rho_{nf} }}\frac{\partial p}{{\partial r}} + v_{nf} \left( {\frac{{\partial^{2} u}}{{\partial r^{2} }} + \frac{1}{r}\frac{\partial u}{{\partial r}} + \frac{{\partial^{2} u}}{{\partial z^{2} }} - \frac{u}{{r^{2} }}} \right) - \sigma_{nf} B_{0}^{2} u, $$

5 $$ u\frac{\partial v}{{\partial r}} + w\frac{\partial v}{{\partial z}} + \frac{vu}{r} = v_{nf} \left( {\frac{{\partial^{2} v}}{{\partial r^{2} }} + \frac{1}{r}\frac{\partial v}{{\partial r}} + \frac{{\partial^{2} v}}{{\partial z^{2} }} - \frac{v}{{r^{2} }}} \right) - \sigma_{nf} B_{0}^{2} v, $$

6 $$ u\frac{\partial w}{{\partial r}} + w\frac{\partial w}{{\partial z}} = - \frac{1}{{\rho_{nf} }}\frac{\partial p}{{\partial z}} + v_{nf} \left( {\frac{{\partial^{2} w}}{{\partial r^{2} }} + \frac{1}{r}\frac{\partial w}{{\partial r}} + \frac{{\partial^{2} w}}{{\partial z^{2} }}} \right), $$

7 $$ u\frac{\partial T}{{\partial r}} + w\frac{\partial T}{{\partial z}} = \alpha_{nf} \left( {\frac{{\partial^{2} T}}{{\partial r^{2} }} + \frac{1}{r}\frac{\partial T}{{\partial r}} + \frac{{\partial^{2} T}}{{\partial z^{2} }}} \right), $$

8 $$ u\frac{\partial a}{{\partial r}} + w\frac{\partial a}{{\partial z}} = D_{A} \left( {\frac{{\partial^{2} a}}{{\partial r^{2} }} + \frac{1}{r}\frac{\partial a}{{\partial r}} + \frac{{\partial^{2} a}}{{\partial z^{2} }}} \right) - k_{c} ab^{2} , $$

9 $$ u\frac{\partial b}{{\partial r}} + w\frac{\partial b}{{\partial z}} = D_{B} \left( {\frac{{\partial^{2} b}}{{\partial r^{2} }} + \frac{1}{r}\frac{\partial b}{{\partial r}} + \frac{{\partial^{2} b}}{{\partial z^{2} }}} \right) + k_{c} ab^{2} , $$

with the corresponding boundary conditions

10 $$ \begin{gathered} u = u_{w} = cr,\, \, v = r\Omega ,w = 0,\, \, T = T_{w} , \, D_{A} \frac{\partial a}{{\partial z}} = k_{s} a,D_{B} \frac{\partial b}{{\partial z}} = - k_{s} a,{\text{ at }}z = 0, \hfill \\ u \to u_{\infty } = ar,\, \, v \to 0, \, T \to T_{\infty } ,a \to a_{0} \,,b \to 0{\text{ as }}z \to \infty . \hfill \\ \end{gathered} $$

The hypothetical relations are characterized as follows:

11 $$  \begin{gathered}   \mu _{{nf}}  = \tfrac{{\mu _{f} }}{{(1 - \phi )^{{2.5}} }},\,{\text{ }}v_{{nf}}  = \tfrac{{\mu _{{nf}} }}{{\rho _{{nf}} }}, \hfill \\   \rho _{{nf}}  = (1 - \phi )\rho _{f}  + \phi \rho _{{CNT}} ,\,{\text{ }}\alpha _{{nf}}  = \tfrac{{k_{{nf}} }}{{\rho _{{nf}} (c_{p} )_{{nf}} }}, \hfill \\   \frac{{k_{{nf}} }}{{k_{f} }} = \frac{{(1 - \phi ) + 2\phi \tfrac{{k_{{CNT}} }}{{k_{{CNT}}  - k_{f} }}\ln (\tfrac{{k_{{CNT}}  + k_{f} }}{{2k_{f} }})}}{{(1 - \phi ) + 2\phi \tfrac{{k_{f} }}{{k_{{CNT}}  - k_{f} }}\ln (\tfrac{{k_{{CNT}}  + k_{f} }}{{2k_{f} }})}}. \hfill \\  \end{gathered}  $$

Using the similarity transformations

12 $$ \begin{aligned}  \eta = z\left( {\frac{c}{{v_{f} }}} \right)^{1/2} , \, u = crf(\eta ), \, v = crg(\eta ), \, w = (c\nu_{f} )^{1/2} h(\eta ), \, \\ \theta (\eta ) = \frac{{T - T_{\infty } }}{{T_{w} - T_{\infty } }}, \, \Psi (\eta ) = \frac{a}{{a_{0} }}, \, \chi (\eta ) = \frac{b}{{b_{0} }}. \\ \end{aligned} $$

The Eqs. (3) to (9) yield

13 $$ \begin{array}{*{20}c} {h^{\prime} + 2f = 0,} \\ {} \\ \end{array} $$

14 $$ \frac{1}{{(1 - \phi )^{2.50} (1 - \phi + \phi \tfrac{{\rho_{CNT} }}{{\rho_{f} }})}}f^{\prime\prime} + g^{2} - f^{2} - hf^{\prime} + \frac{M}{{(1 - \phi + \phi \tfrac{{\rho_{CNT} }}{{\rho_{f} }})}}\left( {\frac{a}{c} - f} \right) + \left( \frac{a}{c} \right)^{2} = 0, $$

15 $$ \frac{1}{{(1 - \phi )^{2.50} (1 - \phi + \phi \tfrac{{\rho_{CNT} }}{{\rho_{f} }})}}g^{\prime\prime} - 2fg - hg^{\prime} - \frac{M}{{(1 - \phi + \phi \tfrac{{\rho_{CNT} }}{{\rho_{f} }})}}g = 0, $$

16 $$ \tfrac{{k_{nf} }}{{k_{f} }}\theta^{\prime\prime} - \Pr \left[ {1 - \phi + \phi \tfrac{{(\rho C_{p} )_{s} }}{{(\rho C_{p} )_{f} }}} \right]h\theta ^{\prime} = 0, $$

17 $$ \Psi ^{\prime\prime} + S_{c} h\Psi ^{\prime} - S_{c} k_{1} \Psi \chi^{2} = 0, $$

18 $$ \delta \chi ^{\prime\prime} - S_{c} h\chi ^{\prime} + S_{c} k_{1} \Psi \chi^{2} = 0, $$

and the boundary condition (10) becomes

19 $$ \begin{aligned} f(0) = & 1,\, \, h(0) = 0,\, \, g(0) = \omega , \, \theta (0) = 1,\, \, \delta \chi ^{\prime}(0) = - k_{2} \Psi (0), \, \Psi ^{\prime}(0) = k_{2} \Psi (0), \, \\ f^{\prime}(\infty ) \to & {\raise0.5ex\hbox{$\scriptstyle a$} \kern-0.1em/\kern-0.15em \lower0.25ex\hbox{$\scriptstyle c$}}, \, g(\infty ) \to 0,\, \, \theta (\infty ) \to 0,\, \, \Psi (\infty ) \to 1, \, \chi (\infty ) \to 0. \\ \end{aligned} $$

For the sake of simplicity, assume $D_{A} = D_{B} = 1,$ *i.e.,* diffusion coefficients are equal ^48^.

20 $$ \Psi (\eta ) + \chi (\eta ) = 1 $$

21 $$ \Psi ^{\prime\prime} - S_{c} h\Psi ^{\prime} - S_{c} k_{1} \Psi (1 - \Psi )^{2} = 0, $$

22 $$ \Psi ^{\prime}(0) = k_{2} \Psi (0), \, \Psi (\infty ) \to 1, $$

The values of varied dimensionless parameters are defined as follows:

23 $$ k_{2} = \tfrac{{k_{s} \sqrt {\nu_{f} } }}{{D_{A} \sqrt c }},\, \, \delta = \tfrac{{D_{A} }}{{D_{B} }},\Pr = \tfrac{{\mu_{f} c_{p} }}{k}{, }M = \tfrac{{\sigma B_{0}^{2} }}{{c\rho_{f} }}, \, \omega = \tfrac{\Omega }{c},k_{1} = \frac{{a_{0}^{2} k_{c} }}{c},S_{c} = \tfrac{{\nu_{f} }}{{D_{A} }}, $$

The skin friction coefficient $C_{fr}$, $C_{g\theta *}$ and the local Nusselt number $Nu_{x}$, are given below

24 $$ \begin{gathered} C_{fr} Re_{r}^{1/2} = \frac{1}{{(1 - \phi )^{2.5} }}f^{\prime}(0),\,C_{{g\theta^{*} }} Re_{r}^{1/2} = \frac{1}{{(1 - \phi )^{2.5} }}g^{\prime}(0), \hfill \\ \;Nu_{r} Re_{r}^{ - 1/2} = - \frac{{k_{nf} }}{{k_{f} }}\theta^{\prime}(0). \hfill \\ \end{gathered} $$

The thermophysical traits of the MWCNTs and the Engine oil are listed in Table 2 and are assumed values are taken as independent of temperature. Table 3 is constructed to make a comparison for varied estimates of $\omega$ by fixing $M = 0.0,2.0,$ with Das et al. ^3^. An excellent concurrence is achieved in this regard.

**Table 2:** Thermophysical attributes of primary fluid (Engine oil) and nanoparticles (MWCNTs) ^49^:

Physical properties	Base fluid	Nanoparticle
	Engine oil	MWCNTs
C_p_(J/kg K)	1910.0	796.00
$\rho$(kg/m^3^)	884.00	1600.0
K(W/mK)	0.1440	3000.0

**Table 3:** Comparison of result $f^{\prime}(0)$ and $- \theta ^{\prime}(0)$ with Das et al. ^3^ for different value of $\omega$ when $k_{1} = k_{2} = \tfrac{a}{c} = 0,{\text{ and }}\Pr = 1.0.$

*M*	$\omega$	$f^{\prime}(0)$		$- \theta ^{\prime}(0)$	
		Das et al. ^3^	Present result	Das et al. ^3^	Present result
**0.0**	0.0	* − *1.1737	* − *1.17399	0.8520	0.85207
	1.0	* − *0.9483	* − *0.94856	0.8757	0.87571
	2.0	* − *0.3263	* − *0.32642	0.9304	0.93040
	5.0	3.1937	3.19371	1.1292	1.12916
**2.0**	0.0	* − *1.8305	* − *1.83048	0.7261	0.72610
	1.0	* − *1.6635	* − *1.66343	0.7422	0.74230
	2.0	* − *1.1754	* − *1.17533	0.7854	0.78540
	5.0	1.8928	1.89296	0.9803	0.98036

## Entropy generation

The nanofluid’s volumetric rate of local entropy generation ^50–52^ in attendance of the magnetic field in attendance of axial symmetry with assumed assumptions is given as under:

25 $$ {S_{gen}^{{\prime\prime\prime}}} = \frac{{k_{nf} }}{{T_{w}^{2} }}\left[ {\left( {\frac{1}{r}\frac{\partial T}{{\partial \theta }}} \right)^{2} + \left( {\frac{\partial T}{{\partial r}}} \right)^{2} + \left( {\frac{\partial T}{{\partial z}}} \right)^{2} } \right] + \frac{{\mu_{nf} }}{{T_{w} }}\Phi + \frac{1}{{T_{w} }}\left( {{\mathbf{J}} - Q{\mathbf{V}}} \right) \cdot ({\mathbf{E}} + {\mathbf{V}} \times {\mathbf{B}}) + \frac{RD}{{a_{0} }}\left( {\frac{\partial a}{{\partial z}}} \right)^{2} + \frac{RD}{{T_{\infty } }}\left( {\frac{\partial a}{{\partial z}}} \right)\left( {\frac{\partial T}{{\partial z}}} \right), $$

26 $$ \begin{gathered} {\mathbf{J}} = \sigma ({\mathbf{E}} + {\mathbf{V}} \times {\mathbf{B}}) \hfill \\ \Phi = 2\left[ {\left( {\frac{1}{r}(\frac{\partial v}{{\partial \theta }} + u)} \right)^{2} + \left( {\frac{\partial u}{{\partial r}}} \right)^{2} + \left( {\frac{\partial w}{{\partial z}}} \right)^{2} } \right] + \left[ {\left( {\frac{1}{r}\frac{\partial w}{{\partial \theta }}} \right) + \left( {\frac{\partial v}{{\partial z}}} \right)} \right]^{2} + \left[ {\left( {\frac{\partial w}{{\partial r}}} \right) + \left( {\frac{\partial u}{{\partial z}}} \right)} \right]^{2} + \left[ {\left( {\frac{1}{r}\frac{\partial u}{{\partial \theta }}} \right) + r\left( \frac{v}{r} \right)_{r} } \right]^{2} , \hfill \\ \end{gathered} $$

It is assumed that the impact of the electric force per unit charge in comparison to ${\mathbf{V}} \times {\mathbf{B}}$ as stated above is ignored. The magnitude of electric current is taken as immensely greater than $Q{\mathbf{V}}.$ Taking into account the aforementioned assumptions, the following is obtained:

27 $$ \begin{gathered} S^{\prime\prime\prime}_{gen} = \frac{{k_{nf} }}{{T_{w}^{2} }}\left( {\frac{\partial T}{{\partial z}}} \right)^{2} + \frac{{\mu_{nf} }}{{T_{w} }}\left\{ \begin{gathered} 2\left[ {\left( {\frac{1}{r}u} \right)^{2} + \left( {\frac{\partial u}{{\partial r}}} \right)^{2} + \left( {\frac{\partial w}{{\partial z}}} \right)^{2} } \right] + \left( {\frac{\partial v}{{\partial z}}} \right)^{2} + \left( {\frac{\partial u}{{\partial z}}} \right)^{2} \hfill \\ \, + \left[ {r\left( \frac{v}{r} \right)_{r} } \right]^{2} \hfill \\ \end{gathered} \right\} + \frac{{\sigma B^{2}_{0} }}{{T_{w} }}\left[ {u^{2} + v^{2} } \right] \hfill \\ \, + \frac{RD}{{a_{0} }}\left( {\frac{\partial a}{{\partial z}}} \right)^{2} + \frac{RD}{{T_{\infty } }}\left( {\frac{\partial a}{{\partial z}}} \right)\left( {\frac{\partial T}{{\partial z}}} \right), \hfill \\ \end{gathered} $$

In Eq. (25), the first, second, third, and fourth, terms signify irreversibility due to heat transfer, the fluid friction irreversibility, the is local entropy generation due to the effect of the magnetic field, and the irreversibility caused by the diffusion effect. The quotient of entropy generation rate $S^{\prime\prime\prime}_{gen}$ and the characteristic entropy generation rate $S^{\prime\prime\prime}_{0}$ is the entropy generation $N_{G}$ and mathematically described as:

28 $$ \begin{gathered} N_{G} = \alpha \theta ^{{\prime}{2}} + Br\left( {\frac{3}{{\text{Re}}}h^{{\prime}{2}} + \mathop r\limits^{\_} \left\{ {(f^{{\prime}{2}} + g^{{\prime}{2}} ) + M(f^{2} + g^{2} )} \right\}} \right) + \frac{\sum }{\Pi }\left( {\Psi ^{{\prime}{2}} + \frac{\theta ^{\prime}\Psi ^{\prime}}{\Pi }} \right), \hfill \\ \alpha = \frac{\Delta T}{{T_{w} }},Br = \frac{{\mu_{nf} c^{2} R^{2} }}{{k_{nf} \Delta T}},{\text{Re}} = \frac{{cR^{2} }}{{\nu_{f} }},\mathop r\limits^{\_} = \frac{r}{R},\sum = \frac{{a_{0} RD}}{k},\Pi = \theta_{w} - 1. \hfill \\ \end{gathered} $$

## Numerical solution

The solution of the Eqs. (13)–(16) and (20) with associated conditions (19) and (22) at the boundary is attained numerically engaging the bvp4c function of MATLAB software. To do so, transform all higher-order equations to the differential equations of order one. The assumed tolerance of the numerical solution is taken as $10^{ - 5}$. To compute the numerical solution, the appropriate estimates of $\eta \to \infty$ namely $\eta = \eta_{\infty } = 3$ is considered by taking into account the values of parameters in the problem.

## Results and discussion

**Figure 2:**
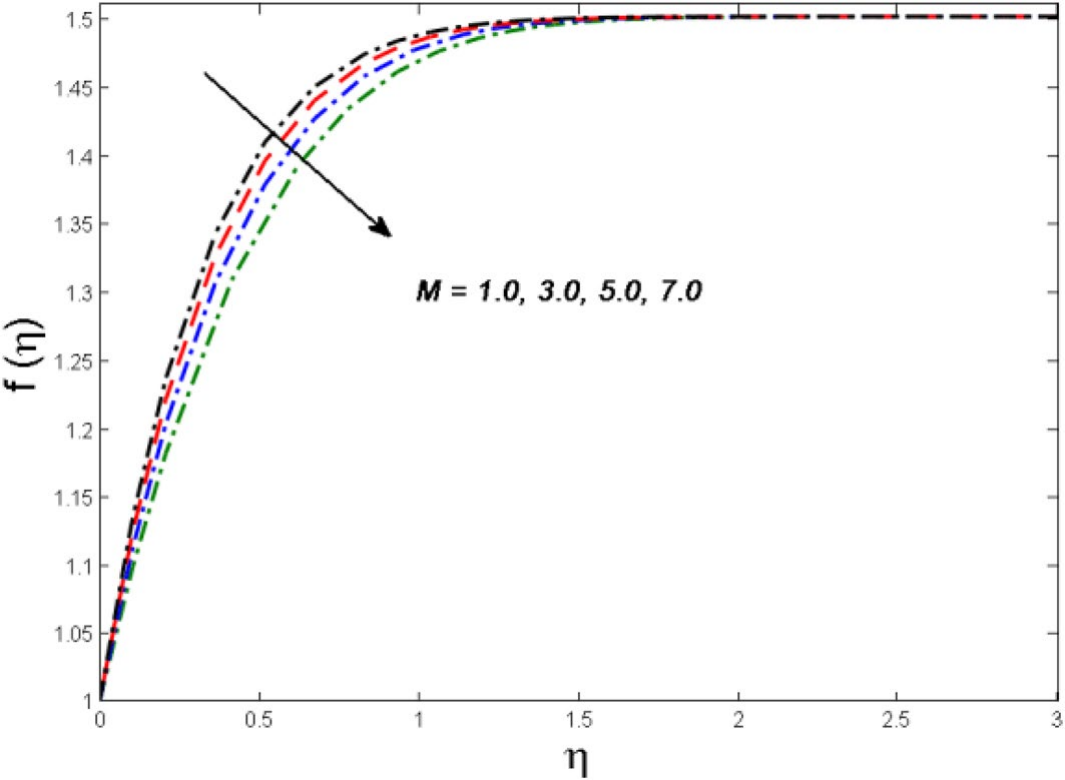
Impact of $M$ on $f(\eta )$.

**Figure 3:**
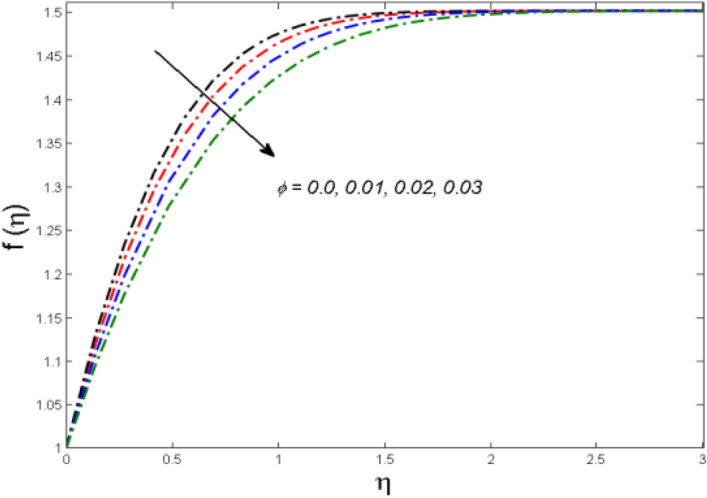
Impact of $\phi$ on $f(\eta )$.

**Figure 4:**
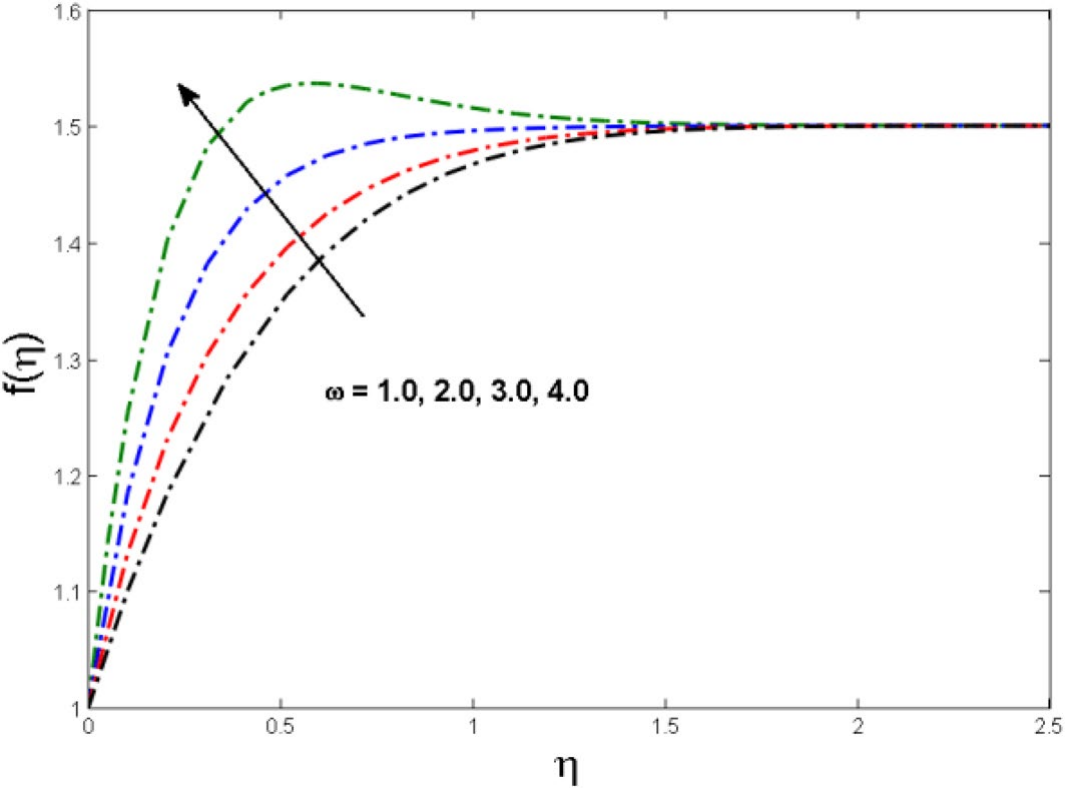
Impact of $\omega$ on $f(\eta )$.

**Figure 5:**
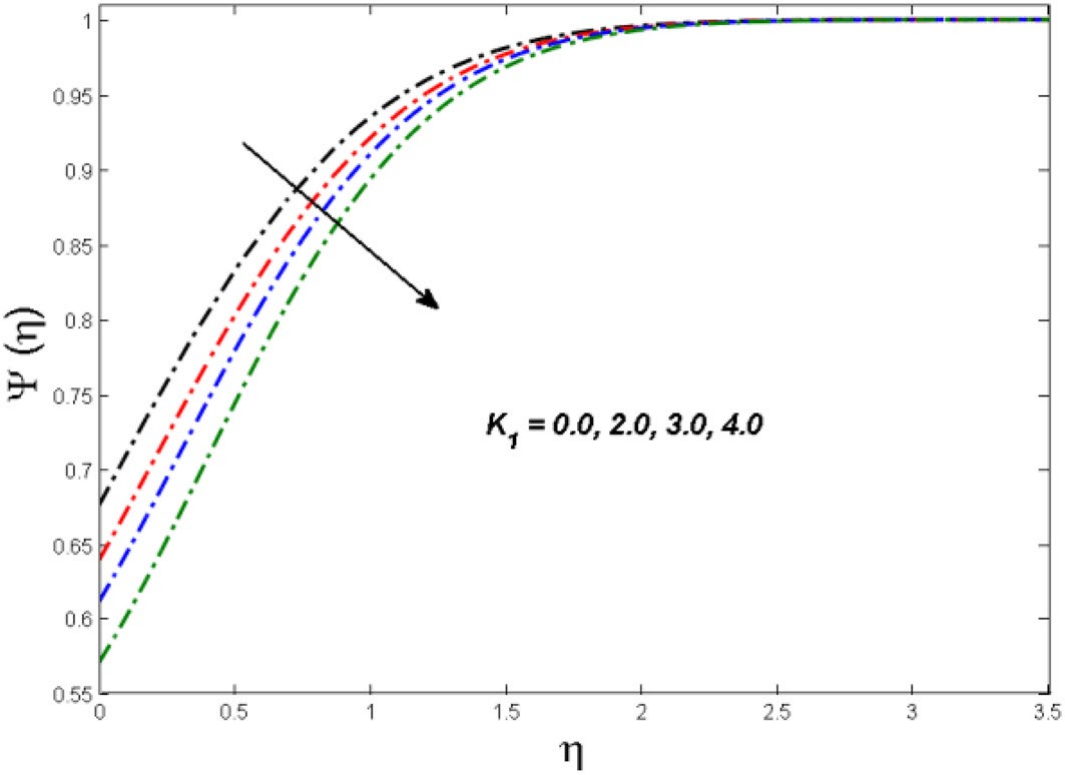
Effect of $K_{1}$ on $\Psi (\eta )$.

**Figure 6:**
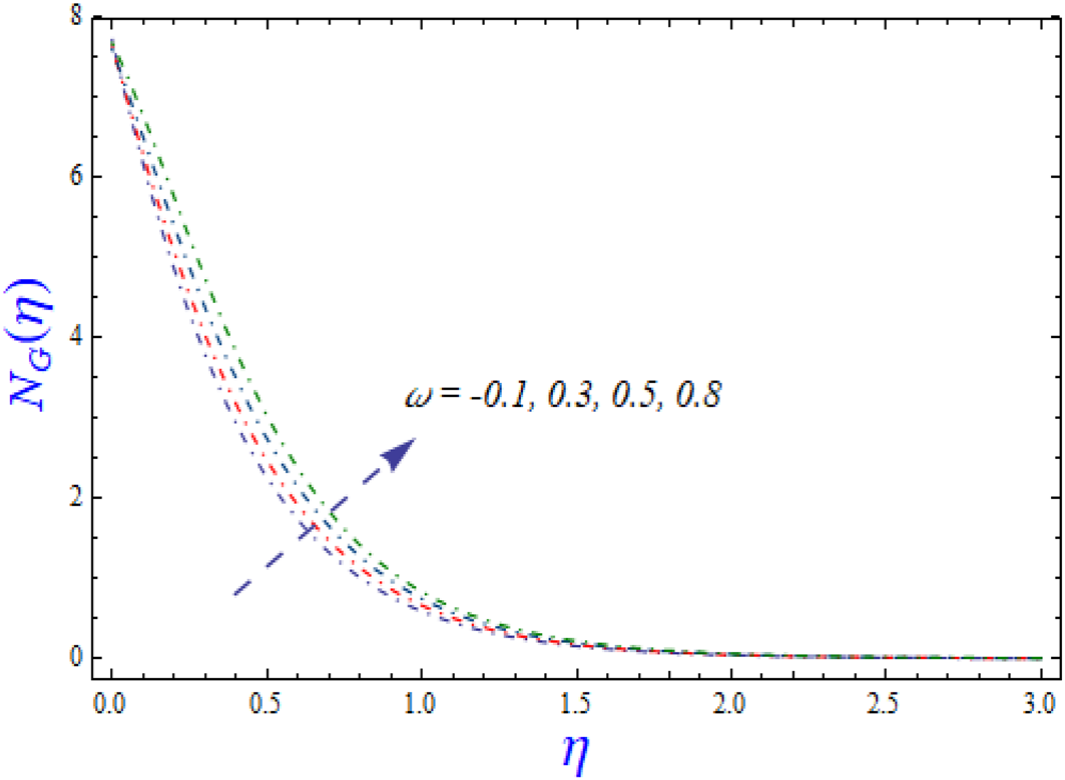
Effect of $\omega$ on $N_{G} (\eta )$.

**Figure 7:**
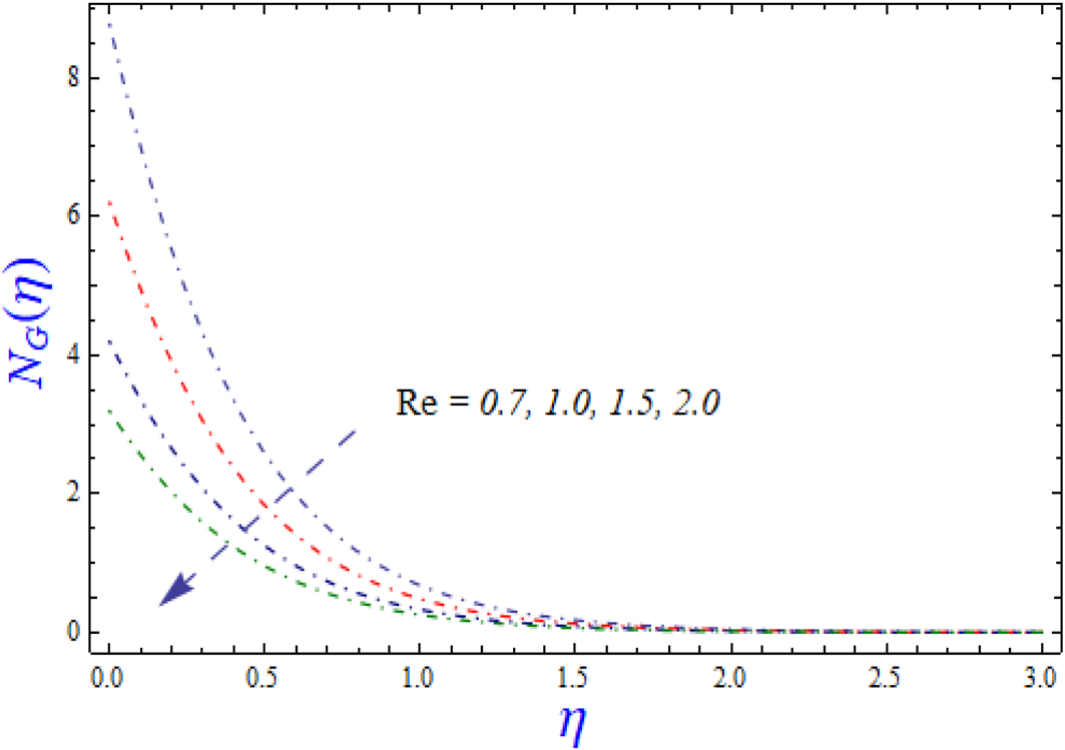
Impact of ${\text{Re}}$ on $N_{G} (\eta )$.

This segment is devoted to the to infer the influences of varied key parameters on the involved distributions. The permissible ranges of the parameters are selected in such a way where the resolution of the graphs is best suited. The acceptable ranges are $1.0 \le M \le 7.0,0.0 \le \phi \le 0.03,0.1 \le \omega \le 4.0,0.0 \le K_{1} \le 4.0,0.7 \le {\text{Re}} \le 2.0.$ The relationship between the fluid velocity along the radial direction with the magnetic parameter $M$ is portrayed in Fig. 2. It is evident from the sketch that the fluid velocity is deteriorated owing to the application of a strong magnetic field. This strong magnetic field fortifies the Lorentz force which works as a resistive force to the fluid flow and eventually fluid velocity is lowered. Figure 3 is drawn to describe the association of the nanoparticle volume fraction $\phi$ and the velocity profile along a radial direction. It is comprehended that the velocity diminishes as nanoparticle volume fraction is augmented. As a matter of fact, it is observed that a high concentration of the nanoparticles will make the fluid more viscous and strengthen the friction drag in the fluid flow. It can be witnessed from the figure that velocity is more in the interval $0.5 \le \eta \le 1.5,$(not exactly measured) and then slows down in the interval $\eta \ge 1.5$ to meet the free stream velocity condition. The correlation between the rotation parameter $\omega$ on the fluid velocity along the radial direction is illustrated in Fig. 4. It is grasped that the fluid velocity is improved once the rotation of the disk is enhanced. Higher estimates of the disk’s rotation result in a strong centrifugal force which pushes the fluid in the outer layer in the radial and tangential directions. Thus, a rapid increase in the momentum boundary layer is seen. The impression of the homogeneous reaction $K_{1}$ on the concentration profile Fig. 5 is drawn. It is clear from the figure that the concentration near the barrier diminishes as estimates of homogeneous reactions are enhanced. This effect is more prominent in the interval $0.0 \le \eta \le 1.3,$ but as $\eta$ is incremented, this impression abates with the strength of the homogeneous reaction. The concentration of the chemical species B is improved when the strength of the homogeneous reaction is enhanced. During this development all reactants are expended, thus a rapid decline in the concentration boundary layer is witnessed. The relationship of the rotation parameter and the Reynolds number with the entropy generation number is depicted in Figs. 6 and 7 respectively. An opposing trend is observed in both illustrations. Higher the rotation of the disk, the more disturbance in the fluid flow owing to the random motion of the molecules. Thus, enhances the disturbance resulting in enriched entropy. Vice versa, on increasing the Reynolds number, a decline in the entropy generation number is witnessed. This is obvious from Eq. (28).

## Experimental design

In mathematical modeling experimental scheme plays a vital role where numerical computational simulation get the better possibility of interpretation. Here data set is generated against the real-world scenarios through computer programming. Since several input parameters may influence the response, an experimental design-based approach called response surface methodology (RSM) for determining which input parameter is influential and which specific level of an input parameter can optimize the response is used. Hence RSM ^38–40^ is used to define the model dependencies of the response variables which are skin friction coefficients ($C_{fr}$, $C_{g\theta *}$) and the local Nusselt number ($Nu_{x}$) with the input parameters which are models parameters including a suction parameter ($\omega$), Nanoparticle volume fraction ($\phi$) and Magnetic parameter ($M$). Notably, it is noticed that Pr is not affecting the skin coefficients while it only impacts local Nusselt number hence it was kept equal to 1 in this study.

Among the several model coefficients and parameters, the sensitivity analysis is also performed, and by using the above-mentioned response variables and parameters of interest only. Besides, only selective inputs scheming parameters that are thought to have an influential variation on the local skin friction coefficients ($C_{fr}$, $C_{g\theta *}$) and the local Nusselt number ($Nu_{x}$) are considered.

By using the standard nonlinear polynomial model experimental design is conducted to assess and evaluate the correlations among the considered response variables and model parameters.

$$ {\text{y}} = {\text{ r}}_{{0}} \, + {\text{ r}}_{{\text{A}}} {\text{A}} + {\text{ r}}_{{\text{B}}} {\text{B}} + {\text{ r}}_{{\text{C}}} {\text{C}} + {\text{ r}}_{{{\text{A}}^{{2}} }} {\text{A}}^{{2}} + {\text{r}}_{{{\text{B}}^{{2}} }} {\text{B}}^{{2}} + {\text{r}}_{{{\text{C}}^{{2}} }} {\text{C}}^{{2}} + {\text{ r}}_{{{\text{A}}B}} {\text{AB }} + {\text{r}}_{{{\text{BC}}}} {\text{BC}} + {\text{r}}_{{{\text{CA}}}} {\text{CA}} + \, \varepsilon . $$

This response surface equation includes an intercept (${\text{r}}_{{0}}$), three linear effects (${\text{r}}_{{\text{A}}} ,{\text{r}}_{{\text{B }}} ,{\text{r}}_{{\text{C}}}$) three quadratic effects (${\text{r}}_{{{\text{A}}^{{2}} }} ,{\text{r}}_{{{\text{B}}^{{2}} }} ,{\text{r}}_{{{\text{C}}^{{2}} }}$), and three interaction effects(${\text{r}}_{{{\text{AB}}}} ,{\text{r}}_{{{\text{BC}}}} ,{\text{r}}_{{{\text{CA}}^{{}} }}$). The response (y) represents the response that is skin friction coefficients ($C_{fr}$, $C_{g\theta *}$) and the local Nusselt number ($Nu_{x}$). For three responses, three response surface equations are considered. For each model parameters A, B and C are Nanoparticle volume fraction ($\phi$), Magnetic parameter ($M$), and suction parameter ($\omega$). For each of these parameters, three levels are low, medium and high levels coded as (− 1, 0, 1) are chosen. The input parameters together with respective notations are presented in Table 4.

**Table 4:** Experimental parameters and their levels.

Parameter	Symbol	Levels		
		Low	Medium	High
$\phi$	A	0.01	0.05	0.09
M	B	0.5	1	1.5
$\omega$	C	0.5	2	3.5

For executing this computational experiment Box-Behnken design is conducted. With number parameters F = 3 and considering number of center points C = 5 the expected number of runs = 2^F^ + 2F + C = 19 were executed. These runs are listed in Table 5.

**Table 5:** Box–Behnken design with 5 center points is presented as real and coded values. Against each combination of parameters the response $C_{fr} Re_{r}^{1/2}$, $C_{{g\theta^{*} }} Re_{r}^{1/2}$ and $\;Nu_{r} Re_{r}^{ - 1/2}$ is also presented.

Runs	Real values			Coded values			Response		
	$\phi$	*M*	$\omega$	*A*	*B*	*C*	$C_{fr} Re_{r}^{1/2}$	$C_{{g\theta^{*} }} Re_{r}^{1/2}$	$\;Nu_{r} Re_{r}^{ - 1/2}$
**1**	0.01	1	3.5	− 1	0	1	1.3293	− 7.3007	1.2049
**2**	0.01	1	0.5	− 1	0	− 1	− 0.5936	− 0.9850	1.1126
**3**	0.05	1	2	0	0	0	0.0394	− 4.2843	1.4555
**4**	0.09	0.5	2	1	− 1	0	0.1402	− 4.3083	1.7973
**5**	0.05	1	2	0	0	0	0.0394	− 4.2843	1.4555
**6**	0.09	1	3.5	1	0	1	1.5428	− 8.3151	1.8701
**7**	0.05	1	2	0	0	0	0.0394	− 4.2843	1.4555
**8**	0.05	0.5	0.5	0	− 1	− 1	− 0.5819	− 0.9813	1.4215
**9**	0.05	1.5	0.5	0	1	− 1	− 0.6785	− 1.1137	1.4122
**10**	0.09	1	0.5	1	0	− 1	0.0477	− 1.1202	1.7414
**11**	0.05	1	2	0	0	0	0.0394	− 4.2843	1.4555
**12**	0.05	0.5	3.5	0	− 1	1	1.5818	− 7.3799	1.5442
**13**	0.05	1	2	0	0	0	0.0394	− 4.2843	1.4555
**14**	0.05	1.5	3.5	0	1	1	1.2942	− 8.1757	1.5144
**15**	0.09	1.5	2	1	1	0	− 0.0362	− 4.8294	1.7767
**16**	0.01	0.5	2	− 1	− 1	0	0.1169	− 3.7739	1.1526
**17**	0.05	1	2	0	0	0	0.0394	− 4.2843	1.4555
**18**	0.01	1.5	2	− 1	1	0	− 0.0450	− 4.2551	1.1381
**19**	0.01	1	3.5	− 1	0	1	1.3293	− 7.3007	1.2049

The model is fitted with a response-surface component. To study the effect considered parameters, the response surface-based analysis of variance (ANOVA) is presented in Table 6. Results include a degree of freedom, the sum of squares (SS), percentage contribution, mean sum of squares (MS), F statistics, and p-value. Based on p-values. It is concluded that the considered parameters suction parameter ($\omega$), Nanoparticle volume fraction ($\phi$), and Magnetic parameter ($M$) have a significant linear impact on all three responses (p-value < 0.001). Similarly, the model parameters have a significant quadratic impact on all responses. Moreover, the model’s interaction effect is significant for $C_{{g\theta^{*} }} Re_{r}^{1/2}$ and $\;Nu_{r} Re_{r}^{ - 1/2}$.

**Table 6:** ANOVA for skin friction coefficients ($C_{fr}$, $C_{g\theta *}$) and the local Nusselt number ($Nu_{x}$) is presented, which includes the quantification and significance of considered linear, interaction, and square model terms.

Source	df	SS	Contribution (%)	MS	F-value	P-value
$${\varvec{C}}_{{{\varvec{fr}}}} {\mathbf{Re}}_{{\varvec{r}}}^{{{\mathbf{1/2}}}}$$						
Linear	3	7.2973	84.65	2.43242	1.30E + 02	< 0.001
Interaction	3	0.0549	0.64	0.0183	9.80E-01	0.449
Square	3	0.9691	11.24	0.32304	1.73E + 01	< 0.001
Residuals	8	0.1494	1.73	0.01868		
Lack of fit	3	0.1494	1.73	0.0498	6.87E + 29	< 0.001
Pure error	5	0	0.00	0		
$${\varvec{C}}_{{{\varvec{g}}\theta \user2{*}}} {\mathbf{Re}}_{{\varvec{r}}}^{{{\mathbf{1/2}}}}$$						
Linear	3	92.034	99.58	30.6779	2.03E + 05	< 0.001
Interaction	3	0.304	0.33	0.1012	6.70E + 02	< 0.001
Square	3	0.081	0.09	0.0269	1.78E + 02	< 0.001
Residuals	8	0.001	0.00	0.0002		
Lack of fit	3	0.001	0.00	0.0004	5.78E + 26	< 0.001
Pure error	5	0	0.00	0		
$${\varvec{Nu}}_{{\varvec{r}}} {\mathbf{Re}}_{{\varvec{r}}}^{{ - {\mathbf{1/2}}}}$$						
Linear	3	0.85585	99.73	0.285284	1.25E + 05	< 0.001
Interaction	3	0.00045	0.05	0.000149	6.52E + 01	< 0.001
Square	3	0.00186	0.22	0.000622	2.73E + 02	< 0.001
Residuals	8	0.00002	0.00	0.000002		
Lack of fit	3	0.00002	0.00	0.000006	1.41E + 26	< 0.001
Pure error	5	0	0.00	0		

**Figure 8:**
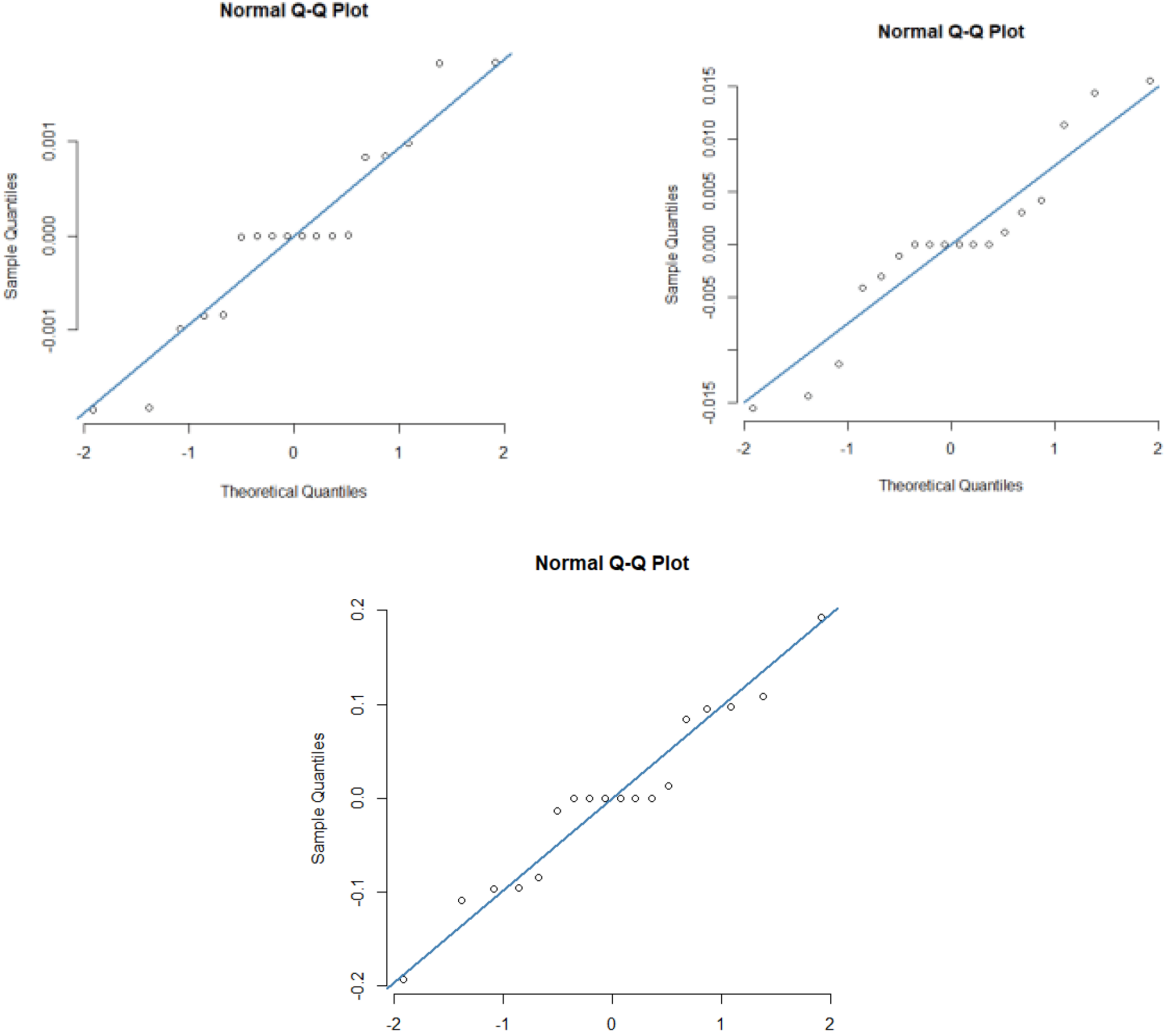
The residual normal quantile–quantile (Q–Q) plot for $C_{fr} Re_{r}^{1/2}$ is presented in the upper left panel, for $C_{{g\theta^{*} }} Re_{r}^{1/2}$ is presented in the upper right panel for $\;Nu_{r} Re_{r}^{ - 1/2}$ is presented in the lower panel.

**Figure 9:**
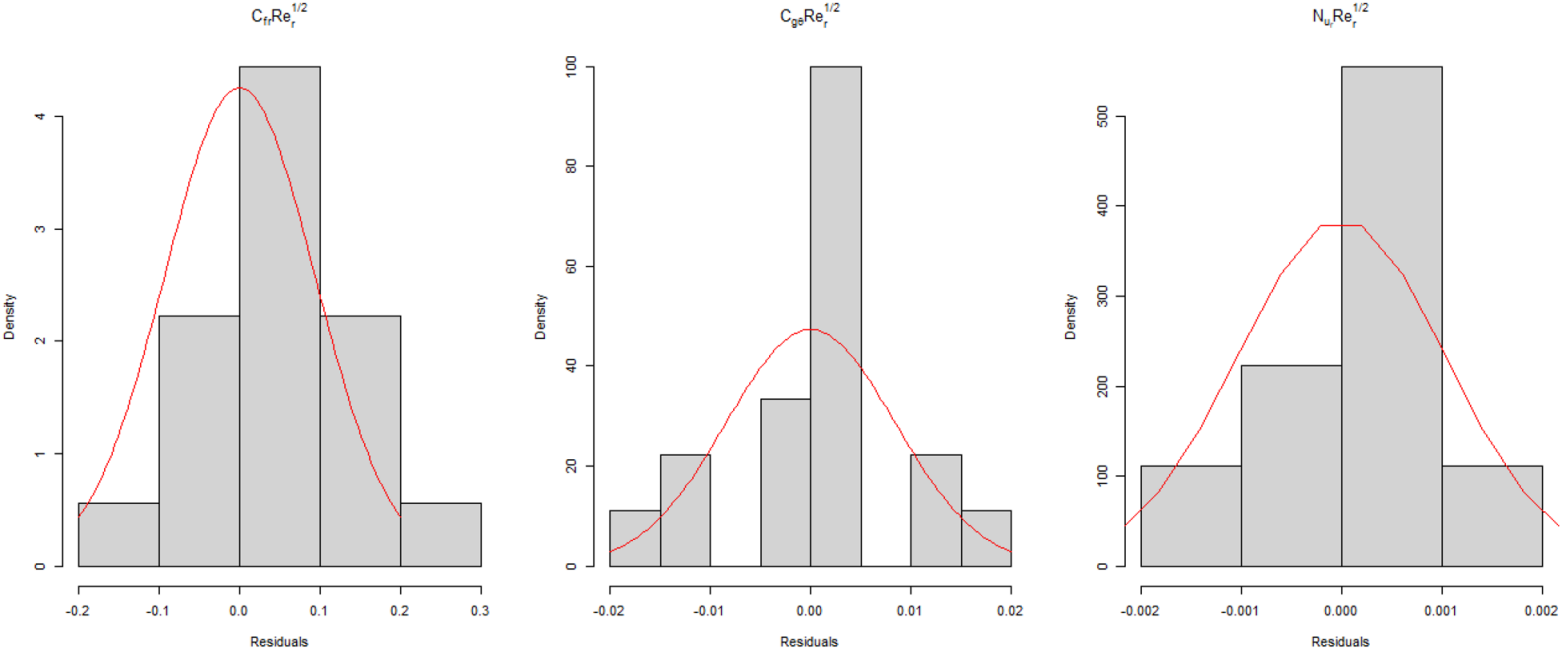
The distribution of residuals from all fitted models is presented here. Indicating all the residuals are normally distributed.

For the goodness of fit purpose, relay on several indicators is considered. For instance, the first one is the lack of fit which has a p-value < 0.001 for all three fitted models. Secondly adjusted R^2^ which presents how much the models explain the variation in response is used. For $C_{fr} Re_{r}^{1/2}$ the adjusted R^2^ is 96.2%, for $C_{{g\theta^{*} }} Re_{r}^{1/2}$ adjusted R^2^ is 99.9% and for $\;Nu_{r} Re_{r}^{ - 1/2}$ adjusted R^2^ is 99.9%. Hence all models explain a very high percentage of the total variation in respective responses. Thirdly, a standard residual quantile–quantile plot is used to evaluate the goodness of fit. A good model that successfully presents the functional relationship between input parameters and response shows one to one relation between theoretical quantiles and observed quantiles. The standard residual quantile–quantile plot for the three fitted models is presented in Fig. 8. This indicates for all three models there is almost one to one relation between theoretical and observed quintiles. Lastly, the residuals of all fitted models are assumed to be normally distributed. The residual distribution of the fitted model is presented in Fig. 9, indicating all three fitted model’s residuals follow a normal distribution. Hence all three models are well-fitted.

To further explore the significant input parameters their estimates together with t-values and p-value is presented in Table 7. This indicates $$\phi$$ (p-value < 0.05), $$\omega$$ (p-value < 0.001) and $$\omega$$ ^2^ (p-value < 0.001) are significant term effecting $$C_{fr} Re_{r}^{1/2}$$. Similarly $$\phi$$*,* *M,* $$\omega$$*,* $$\phi$$$$\omega$$*,* *M *$$\omega$$*,* $$\phi$$^s^ and $$\omega$$ ^2^ are significantly (p-value < 0.001) effecting $$C_{{g\theta^{*} }} Re_{r}^{1/2}$$ and $$\;Nu_{r} Re_{r}^{ - 1/2}$$.

**Table 7:** Fitted model terms estimates mates together with t-values and p-value is presented.

Term	Estimate	Std	t-value	p-value
$${\varvec{C}}_{{{\varvec{fr}}}} {\mathbf{Re}}_{{\varvec{r}}}^{{{\mathbf{1/2}}}}$$				
Intercept	0.03942	0.055792	0.7066	0.499894
A ($\phi$)	0.11086	0.048317	2.2945	0.040909
B (*M)*	− 0.09031	0.048317	− 1.8692	0.098534
C ($\omega$)	0.94430	0.048317	19.5439	< 0.001
AB ($\phi$ *M)*	− 0.00364	0.068331	− 0.0533	0.958788
AC ($$\phi$$$$\omega$$)	− 0.10694	0.068331	− 1.565	0.156208
BC (*M* $\omega$)	− 0.04773	0.068331	− 0.6985	0.504659
A^2^ ($\phi$ ^2^)	0.09109	0.065422	1.3924	0.201281
B^2^*(M*^*2*^*)*	− 0.08656	0.065422	− 1.3231	0.222372
C^2^($\omega$ ^2^)	0.45103	0.065422	6.8942	< 0.001

Term	Estimate	Std	Error	t
$${\varvec{C}}_{{{\varvec{g}}\theta \user2{*}}} {\mathbf{Re}}_{{\varvec{r}}}^{{{\mathbf{1/2}}}}$$				
Intercept	− 4.28430	0.005018	− 853.758	< 0.001
A ($\phi$)	− 0.28228	0.004346	− 64.9541	< 0.001
B (*M)*	− 0.24130	0.004346	− 55.5237	< 0.001
C ($\omega$)	− 3.37140	0.004346	− 775.772	< 0.001
AB ($\phi$ *M)*	− 0.00999	0.006146	− 1.6255	0.14272
AC ($$\phi$$$$\omega$$)	− 0.21980	0.006146	− 35.7628	< 0.001
BC (*M* $\omega$)	− 0.16585	0.006146	− 26.9849	< 0.001
A^2^ ($\phi$ ^2^)	− 0.01248	0.005884	− 2.1203	< 0.001
B^2^*(M*^*2*^*)*	0.00510	0.005884	0.867	0.41119
C^2^($\omega$ ^2^)	− 0.13344	0.005884	− 22.6773	< 0.001
$${\varvec{Nu}}_{{\varvec{r}}} {\mathbf{Re}}_{{\varvec{r}}}^{{ - {\mathbf{1/2}}}}$$				
Intercept	1.45550	0.000616	2362.915	< 0.001
A ($\phi$)	0.32216	0.000533	603.9211	< 0.001
B (*M)*	− 0.00928	0.000533	− 17.3868	< 0.001
C ($\omega$)	0.05573	0.000533	104.4847	< 0.001
AB ($\phi$ *M)*	− 0.00153	0.000754	− 2.0214	0.001
AC ($$\phi$$$$\omega$$)	0.00910	0.000754	12.0623	< 0.001
BC (*M* $\omega$)	− 0.00513	0.000754	− 6.7934	< 0.001
A^2^ ($\phi$ ^2^)	0.00992	0.000722	13.7409	< 0.001
B^2^*(M*^*2*^*)*	0.00075	0.000722	1.0384	0.312
C^2^($\omega$ ^2^)	0.01682	0.000722	23.2938	< 0.001

The fitted models are

$$ \begin{aligned} C_{fr} Re_{r}^{1/2} &= 0.0{3942} + 0.{11}0{\text{86A }} - \, 0.0{9}0{3}0{\text{B}} + 0.{\text{9443C}} - 0.00{\text{36AB }} - 0.{1}0{\text{694AC }} \\ &\quad- 0.0{\text{4773BC}} + 0.0{91}0{\text{9A}}^{{2}} - 0.0{\text{8656B}}^{{{2 } + }} 0.{451}0{\text{33 C}}^{{2}} . \end{aligned} $$

$$ \begin{aligned} C_{{g\theta^{*} }} Re_{r}^{1/2} &= - {4}.{2843}0 - 0.{\text{28228A}} - 0.{2413}0{\text{B}} - {3}.{3714}0{\text{C}} - 0.00{\text{999AB}} \\ &\quad- 0.{2198}0{\text{AC}} - 0.{\text{16585BC}} - 0.0{\text{1248A}}^{{2}} + 0.00{51}0{\text{B}}^{{2}} - 0.{\text{13344 C}}^{{2}} , \end{aligned} $$

$$ \begin{aligned} Nu_{r} Re_{r}^{ - 1/2} &= {1}.{4555}0 + 0.{\text{32216A}} - 0.00{\text{928B}} + 0.0{\text{5573C}} - 0.00{\text{153AB}} \\ &\quad+ 0.00{91}0{\text{AC}} - 0.00{\text{513BC}} + 0.00{\text{992A}}^{{{2} + }} 0.000{\text{75B}}^{{2}} + 0.0{\text{1682 C}}^{{2}} . \end{aligned} $$

Sensitivity in terms of model variables is commonly defined as a derivation of the response function. Sensitivity analysis explores the peculiar prerequisites provided by model output allocated by input variables, compared to model vigor estimation. Hence, the sensitivity functions of input parameters A ($\phi$**),** B (***M),*** and C ($\omega$**)** are expressed as the partial derivative of response as

$$ \frac{{\partial {\text{(C}}_{{{\text{fr}}}} {\text{Re}}_{r}^{1/2} )}}{\partial A} = {0}{\text{.11086 + 0}}{\text{.18219A}} - {0}{\text{.00364B}} - {0}{\text{.10694C,}} $$

$$ \frac{{\partial {\text{(C}}_{{{\text{fr}}}} {\text{Re}}_{r}^{1/2} )}}{\partial B} = - {0}{\text{.09031}} - {0}{\text{.00364A}} - {0}{\text{.17312B}} - {0}{\text{.04773C,}} $$

$$ \frac{{\partial {\text{(C}}_{{{\text{fr}}}} {\text{Re}}_{r}^{1/2} )}}{\partial C} = {0}{\text{.94430}} - {0}{\text{.10694A}} - {0}{\text{.04773B + 0}}{\text{.90207C,}} $$

$$ \frac{{\partial {\text{(C}}_{{{\text{g}}\theta }} {\text{Re}}_{r}^{1/2} )}}{\partial A} = - {0}{\text{.28228}} - {0}{\text{.02495A}} - {0}{\text{.00999B}} - {0}{\text{.21980C,}} $$

$$ \frac{{\partial {\text{(C}}_{{{\text{g}}\theta }} {\text{Re}}_{r}^{1/2} )}}{\partial B} = - {0}{\text{.24130}} - {0}{\text{.00999A + 0}}{\text{.01020B}} - {0}{\text{.16585C,}} $$

$$ \frac{{\partial {\text{(C}}_{{{\text{g}}\theta }} {\text{Re}}_{r}^{1/2} )}}{\partial C} = { - 3}{\text{.37140}} - {0}{\text{.21980A}} - {0}{\text{.16585B}} - {0}{\text{.26688C,}} $$

$$ \frac{{\partial {\text{(N}}_{{{\text{u}}_{{\text{r}}} }} {\text{Re}}_{r}^{1/2} )}}{\partial A} = {0}{\text{.32216 + 0}}{\text{.01985A}} - {0}{\text{.00153B + 0}}{\text{.00910C,}} $$

$$ \frac{{\partial {\text{(N}}_{{{\text{u}}_{{\text{r}}} }} {\text{Re}}_{r}^{1/2} )}}{\partial B} = - {0}{\text{.00928}} - {0}{\text{.00153A + 0}}{\text{.00150B}} - {0}{\text{.00513C,}} $$

$$ \frac{{\partial {\text{(N}}_{{{\text{u}}_{{\text{r}}} }} {\text{Re}}_{r}^{1/2} )}}{\partial C} = 0.{05574 + 0}{\text{.00910A}} - {0}{\text{.00513B + 0}}{\text{.03365C}}{.} $$

**Figure 10:**
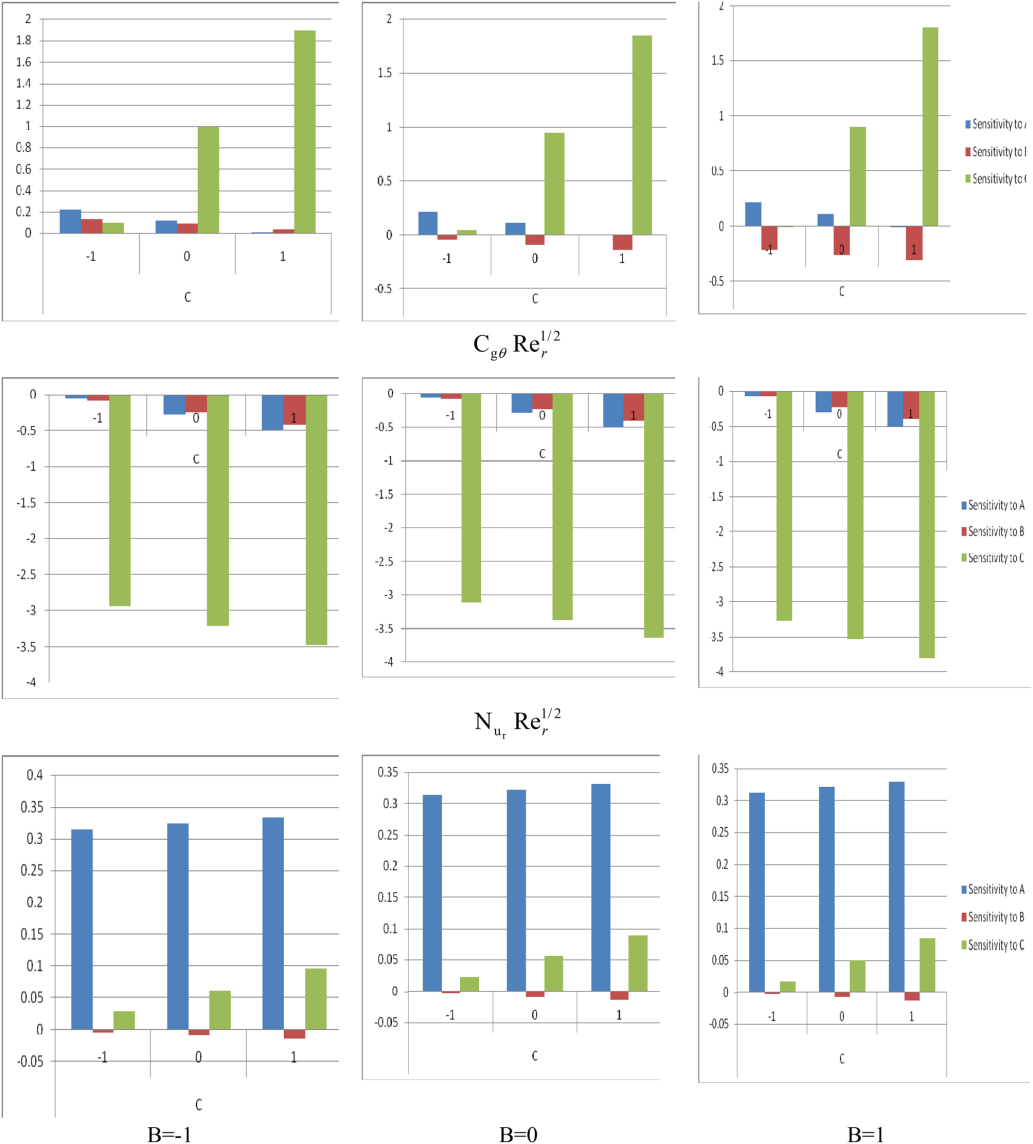
Sensitivity analysis with A = 0 and all combinations of Magnetic parameter and suction parameter for skin friction coefficients $C_{fr} Re_{r}^{1/2}$ is presented in the upper panel, for skin coefficient $C_{{g\theta^{*} }} Re_{r}^{1/2}$ is presented in the middle panel and for the local Nusselt number $\;Nu_{r} Re_{r}^{ - 1/2}$ is presented in the lower panel.

By using the above partial derivatives for skin friction coefficients $C_{fr} Re_{r}^{1/2}$ and $C_{{g\theta^{*} }} Re_{r}^{1/2}$ , and for the local Nusselt number $\;Nu_{r} Re_{r}^{ - 1/2}$ respective sensitivity measures are extracted. These sensitivity measures with A = 0 and all combinations of Magnetic parameters and C are presented in Table 8. The sensitivity measures with positive values indicate the increase of input parameters ($\omega$,$\phi$ and $M$) with the increase of response and vice versa with the sensitivity measures having a negative value. The findings of sensitivity analysis are also presented graphically in Fig. 10 for better understanding. Here, it is used bar plot positive sensitivity measures are presented by upward bars while negative sensitivity measures are presented by downward bars. The upper panel of Fig. 10 presents the sensitivity analysis with A = 0 and all combinations of Magnetic parameters and C for skin friction coefficients $C_{fr} Re_{r}^{1/2}$. With the lower level of the Magnetic parameter and all levels of C, the skin friction coefficient $C_{fr} Re_{r}^{1/2}$ has positive sensitivity. With the middle and upper level of the Magnetic parameter, skin fraction coefficient $C_{fr} Re_{r}^{1/2}$ sensitivity to Nanoparticle volume fraction and the suction parameter is positive and it shows negative sensitivity to the Magnetic parameter. For all levels of the Magnetic parameter at C = 1, the skin friction coefficient $C_{fr} Re_{r}^{1/2}$ is highly sensitive to the suction parameter. The middle panel of Fig. 10 presents the sensitivity analysis with Nanoparticle volume fraction = 0 and all combination of Magnetic parameter and suction parameter for skin friction coefficients $C_{{g\theta^{*} }} Re_{r}^{1/2}$. At all considered levels of Nanoparticle volume fraction, Magnetic parameter, and suction parameter skin fraction coefficient $C_{{g\theta^{*} }} Re_{r}^{1/2}$ shows negative sensitivity to Nanoparticle volume fraction, Magnetic parameter, and suction parameter. Within different levels of the Magnetic parameter, similar sensitivity behaviors are observed. It appears the skin friction coefficient $C_{{g\theta^{*} }} Re_{r}^{1/2}$ is least sensitive to the Nanoparticle volume fraction. Similarly, the skin friction coefficient $C_{{g\theta^{*} }} Re_{r}^{1/2}$ is highly sensitive to the suction parameter. The lower panel of Fig. 10 presents the sensitivity analysis with A = 0 and all combination of Magnetic parameter and suction parameter for the local Nusselt number $\;Nu_{r} Re_{r}^{ - 1/2}$. It appears at all considered levels of Magnetic parameter and suction parameter the sensitivity of local Nusselt number $\;Nu_{r} Re_{r}^{ - 1/2}$ towards Nanoparticle volume fraction and the suction parameter is positive while it is negative towards the Magnetic parameter. Its positive sensitivity towards the suction parameter increases with the increase of levels of a suction parameter. Similarly, the negative sensitivity towards the Magnetic parameter increases towards the negative as the level of suction parameter increases. Moreover, it is highly sensitive to Nanoparticle volume fraction at all levels of the Magnetic parameter and suction parameter.

**Table 8:** Sensitivity analysis with A = 0 and all combination of magnetic parameter and suction parameter for skin friction coefficients $C_{fr} Re_{r}^{1/2}$ , $C_{{g\theta^{*} }} Re_{r}^{1/2}$ and the local Nusselt number $\;Nu_{r} Re_{r}^{ - 1/2}$ is presented.

B	C	Sensitivity								
		$\frac{{\partial {\text{(C}}_{{{\text{fr}}}} {\text{Re}}_{r}^{1/2} )}}{\partial A}$	$\frac{{\partial {\text{(C}}_{{{\text{fr}}}} {\text{Re}}_{r}^{1/2} )}}{\partial B}$	$\frac{{\partial {\text{(C}}_{{{\text{fr}}}} {\text{Re}}_{r}^{1/2} )}}{\partial B}$	$\frac{{\partial {\text{(C}}_{{{\text{g}}\theta }} {\text{Re}}_{r}^{1/2} )}}{\partial A}$	$\frac{{\partial {\text{(C}}_{{{\text{g}}\theta }} {\text{Re}}_{r}^{1/2} )}}{\partial B}$	$\frac{{\partial {\text{(C}}_{{{\text{g}}\theta }} {\text{Re}}_{r}^{1/2} )}}{\partial C}$	$\frac{{\partial {\text{(N}}_{{{\text{u}}_{{\text{r}}} }} {\text{Re}}_{r}^{1/2} )}}{\partial A}$	$\frac{{\partial {\text{(N}}_{{{\text{u}}_{{\text{r}}} }} {\text{Re}}_{r}^{1/2} )}}{\partial B}$	$\frac{{\partial {\text{(N}}_{{{\text{u}}_{{\text{r}}} }} {\text{Re}}_{r}^{1/2} )}}{\partial C}$
− 1	− 1	0.221444	0.130533	0.089967	− 0.05249	− 0.08565	− 2.93867	0.314588	− 0.00565	0.027213
	0	0.114505	0.082804	0.992033	− 0.27229	− 0.2515	− 3.20555	0.323688	− 0.01078	0.060863
	1	0.007566	0.035075	1.894099	− 0.49209	− 0.41735	− 3.47243	0.332788	− 0.0159	0.094513
0	− 1	0.217801	− 0.04258	0.042238	− 0.06248	− 0.07545	− 3.10452	0.313063	− 0.00415	0.022088
	0	0.110862	− 0.09031	0.944304	− 0.28228	− 0.2413	− 3.3714	0.322163	− 0.00928	0.055738
	1	0.003923	− 0.13804	1.84637	− 0.50208	− 0.40715	− 3.63828	0.331263	− 0.0144	0.089388
1	− 1	0.214158	− 0.2157	− 0.00549	− 0.07247	− 0.06525	− 3.27036	0.311538	− 0.00265	0.016963
	0	0.107219	− 0.26343	0.896575	− 0.29227	− 0.23109	− 3.53725	0.320638	− 0.00778	0.050613
	1	0.00028	− 0.31116	1.798641	− 0.51207	− 0.39694	− 3.80413	0.329738	− 0.0129	0.084263

**Figure 11:**
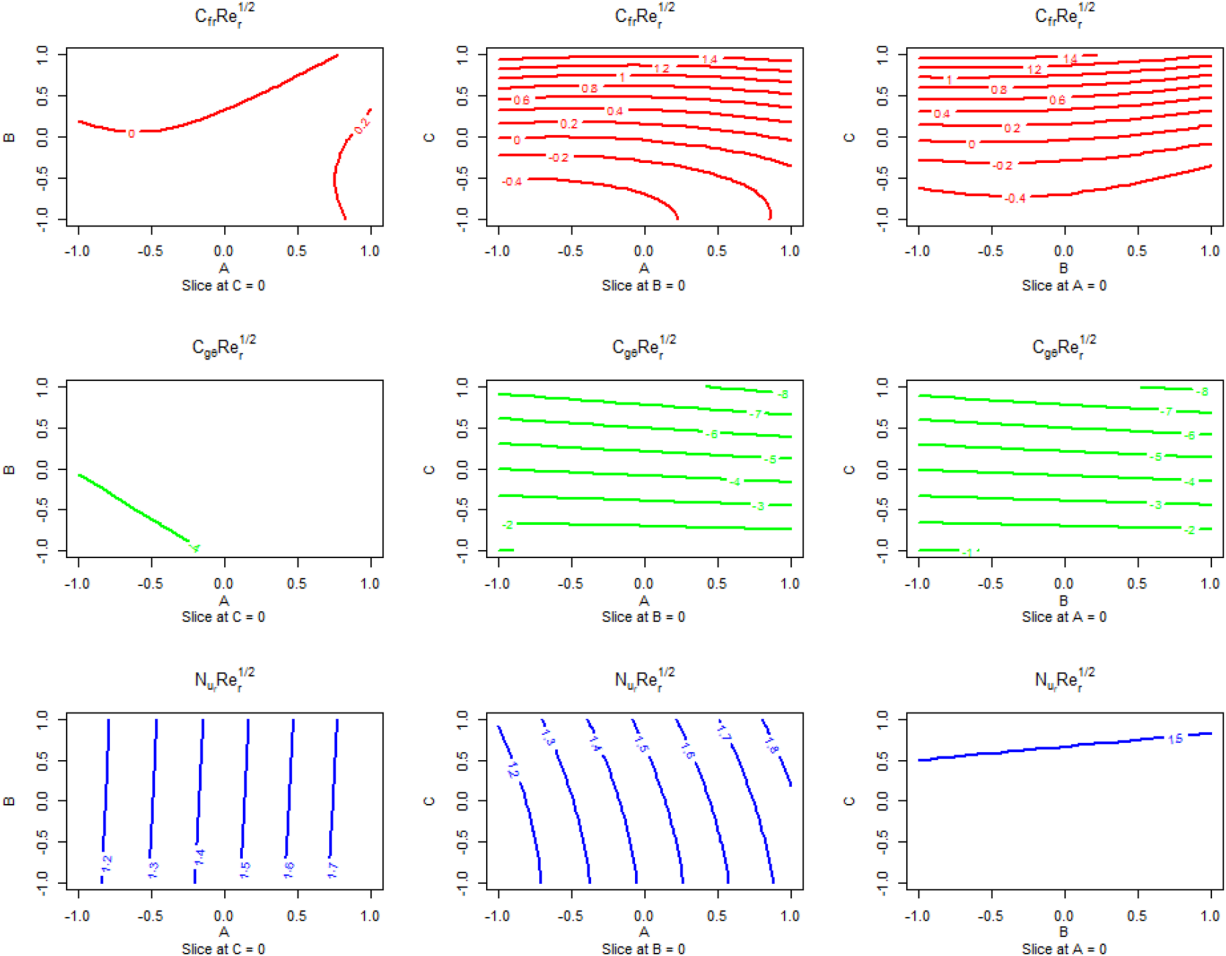
Predicted responses as a function of input parameters coded level with Nanoparticle volume fraction and Magnetic parameter, Magnetic parameter, and suction parameter and Nanoparticle volume fraction and suction parameter are presented for skin friction coefficients $C_{fr} Re_{r}^{1/2}$ in the upper panel, for skin coefficient $C_{{g\theta^{*} }} Re_{r}^{1/2}$ in the middle panel and for the local Nusselt number $\;Nu_{r} Re_{r}^{ - 1/2}$ in the lower panel.

Predicted responses as a function of the input parameter’s levels Nanoparticle volume fraction and Magnetic parameter, Magnetic parameter and suction parameter, and Nanoparticle volume fraction and suction parameter are presented in Fig. 11. The upper panel presents the predicted responses for skin friction coefficients $C_{fr} Re_{r}^{1/2}$. When the effect of the suction parameter is kept zero then the skin friction coefficient maximizes at the highest level of Nanoparticle volume fraction and Magnetic parameter. When the effect of the Magnetic parameter kept zeroing the skin friction coefficient maximizes at a high level of a suction parameter with any level of Magnetic parameter. When the effect of the Nanoparticle volume fraction kept zeroing the skin coefficient maximizes at a high level of the suction parameter with any level of Nanoparticle volume fraction. The middle panel presents the predicted responses for skin friction coefficients $C_{{g\theta^{*} }} Re_{r}^{1/2}$. When the effect of the suction parameter is kept zero then the skin coefficient minimizes at the middle level of Nanoparticle volume fraction and Magnetic parameter. When the effect of the Magnetic parameter kept zeroing the skin coefficient minimizes at a lower level of suction parameter and Nanoparticle volume fraction. When the effect of the Nanoparticle volume fraction kept zeroing the skin coefficient minimizes at a lower level of suction parameter and Magnetic parameter. The lower panel presents the predicted responses for the local Nusselt number $\;Nu_{r} Re_{r}^{ - 1/2}$. When the effect of the suction parameter is kept zero then the local Nusselt number maximizes are high level of Nanoparticle volume fraction and any level of Magnetic parameter. When the effect of the Magnetic parameter kept zeroing the local Nusselt number maximizes at a higher level of suction parameter and a higher level of Nanoparticle volume fraction. When the effect of the Nanoparticle volume fraction kept zeroing the local Nusselt number maximizes at a higher level of Magnetic parameter and does not affect the change of a suction parameter.

## Concluding remarks

In this study, we have analyzed the flow of magnetohydrodynamic nanofluid containing MWCNTs submerged into the engine oil over a rotating disk. The concentration equation is modified by considering the homogeneous-heterogeneous reactions. The assistance of the bvp4c numerical technique combined with the response surface methodology is obtained for the solution of a highly nonlinear system of equations. The sensitivity analysis is performed using a response surface methodology. This is a unique problem in its domain that has been examined with numerical and statistical techniques.

Based on the results, the following findings are devised:

- Based on the normal quantile–quantile residual plot, adjusted R^2^, and hypothesis testing though p-value, all three fitted models for skin friction coefficients $C_{fr} Re_{r}^{1/2}$ and $C_{{g\theta^{*} }} Re_{r}^{1/2}$, and for the local Nusselt number $\;Nu_{r} Re_{r}^{ - 1/2}$ show the best fit.
- All three modeled responses residuals for skin friction coefficients and the local Nusselt number are approximately normal and symmetric.
- The skin friction coefficient $C_{fr} Re_{r}^{1/2}$ and local Nusselt number $\;Nu_{r} Re_{r}^{ - 1/2}$ are positively sensitive to nanoparticle volume fraction while it is high positively sensitive to the suction parameter. It is negatively sensitive to the Magnetic parameter.
- The skin friction coefficient $C_{{g\theta^{*} }} Re_{r}^{1/2}$ is negatively sensitive to all input parameters.
- The skin friction coefficient $C_{fr} Re_{r}^{1/2}$ is optimized on a higher level of input parameters.
- The skin friction coefficient $C_{{g\theta^{*} }} Re_{r}^{1/2}$ is optimized at a higher level of Nanoparticle volume fraction and a higher level of a magnetic parameter.
- The local Nusselt number $\;Nu_{r} Re_{r}^{ - 1/2}$ is optimized at a higher level of magnetic parameter and a higher level of a suction parameter.
- The fluid velocity deteriorates for the high estimates of the nanoparticle volume fraction.

## Abbreviations

*B*: Magnetic field strength
$B_{0}$: Constant magnetic flux density
*C*_*f*_: Skin friction coefficient
*D*_*A,*_* D*_*B*_: Diffusion coefficients
*k*_*c*_: Volumetric rate of local entropy Generation
*k*_*s*_: Strength of heterogeneous reaction
***k***_***f***_: Thermal conductivities of fluid
***k***_***CNT***_: Thermal conductivities of nanomaterial
$$M$$: Magnetic parameter
*Nu*_r_: Nusselt number
$$N_{G}$$: Entropy generation
p: Pressure
Re: Local Reynolds number
***S***_***c***_: Schmidt number
$S_{gen}^{{\prime\prime}^{\prime}}$: Volumetric rate of local entropy Generation
$S_{0}^{{\prime\prime}^{\prime}}$: The characteristic entropy generation rate
*T*: Temperature
*T*_*∞*_: Ambient temperature
***u***: Velocity component in the radial direction
*v*: Velocity component in the tangential direction
w: Velocity component in the axial direction
*z*: Normal direction in cylindrical polar coordinates

## Greek symbols

$\alpha$: Dimensionless temperature difference
$\alpha_{nf}$: Nanofluid thermal diffusivity
$$\delta$$: Pressure
$\eta$: A scaled boundary -layer coordinate
$\mu_{f}$: Fluid dynamic viscosity
$\mu_{nf}$: Nanofluid dynamic viscosity
$$\Pi$$: Temperature difference
$$\sigma$$: Constant parameter
$\sum$: Viscous dissipation function
$\phi$: Nanoparticle volume fraction
$\Phi$: Angular velocity of the disk
$$\Psi$$: Nanoparticle concentration
$$\omega$$: Suction parameter
$\Omega$: Surface shear stress
